# Supplementary material for: A whole-animal phenotypic drug screen identifies suppressors of atherogenic lipoproteins
Source: eLife. 2026 Aug 27;14:RP105314. doi: 10.7554/eLife.105314 (PMC13521566; doi:10.7554/eLife.105314)
Supplement: Supplementary file 2 [file elife-105314-code1.zip › JHDL_primary_result/CETRIMONIUM BROMIDE/CETRIMONIUM BROMIDE_JHDL_9_036_036_2019_06_28_11_39.pdf]

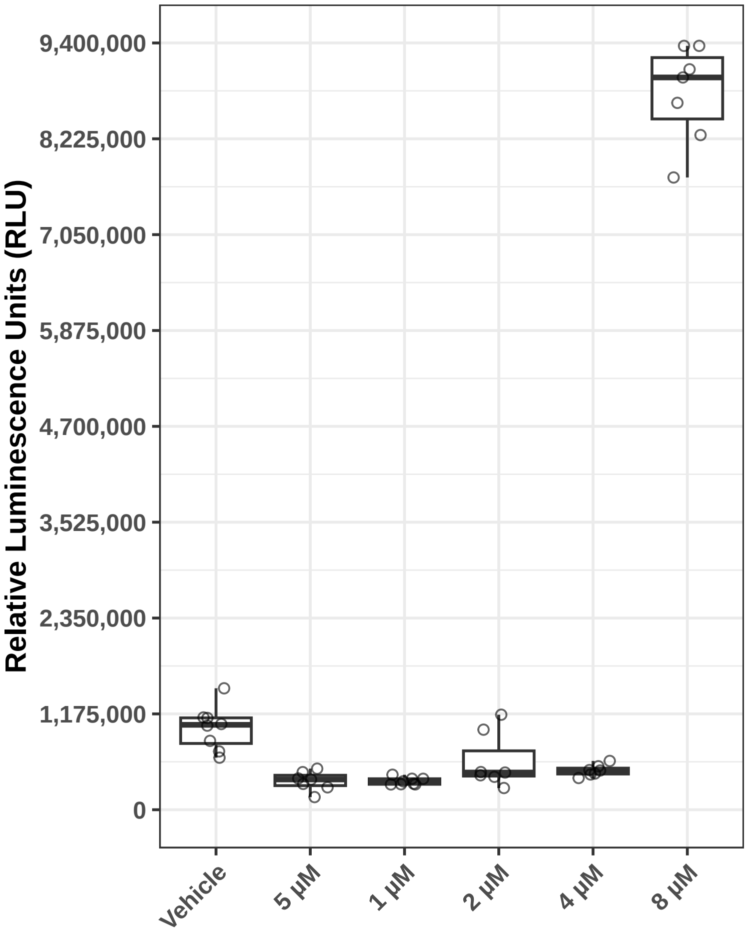
Relative Luminescence Units (RLU)
9,400,000
8,225,000
7,050,000
5,875,000
4,700,000
3,525,000
2,350,000
1,175,000
0
Vehicle
5 μM
1 μM
2 μM
4 μM
8 μM
